# Management of Complex Hand Injuries with Suspension Interposition Arthroplasty and Carpometacarpal Joint Buttress Plating Based on Intra-operative Creativity and Flexibility: A Case Report

**DOI:** 10.5704/MOJ.2111.020

**Published:** 2021-11

**Authors:** SS Mooi, RF Muhammad-Nawawi

**Affiliations:** Department of Orthopaedics, Hospital Kuala Lumpur, Kuala Lumpur, Malaysia

**Keywords:** trapezoidectomy, suspension interposition arthroplasty, carpometacarpal joint buttress plating

## Abstract

A young patient presented to our centre with swollen right hand following a motor vehicle accident. He was diagnosed with closed fractures of trapezoid, ulnar three metacarpal bones, radial styloid and ulnar styloid. The hand injuries were complicated with compartment syndrome. Emergent fasciotomy and application of external fixator of the hand were performed. Definitive fixation of the fractures was delayed due to the wound care post fasciotomy. During the definitive fixation of the hand, the trapezoid was found to be comminuted and completely extruded. Abundant callus was found at the fracture sites of the metacarpal bones. Anatomic fixation was not feasible. Principle-based intra-operative creativity and flexibility were of great significance in the unconventional fixation of the complex hand injuries described in this case report.

## Introduction

The trapezoid is the least commonly fractured carpal bone due to its well-protected position in the wrist. High-energy bending or axial force is required to fracture the trapezoid. Undisplaced fracture is usually treated conservatively. Fracture with large fragments can be treated with open reduction and internal fixation (ORIF). Trapezoid fracture with comminution which is not fixable with an internal implant is treated with carpometacarpal arthrodesis^1^.

Isolated carpometacarpal joint fracture/dislocation is common in the little finger. However, carpometacarpal joint fracture/dislocation of ring finger is uncommon. Similarly, multiple carpometacarpal fracture-dislocations are not common. Treatment of these high-energy injuries requires ORIF^2^.

## Case Report

**Fig. 1:**
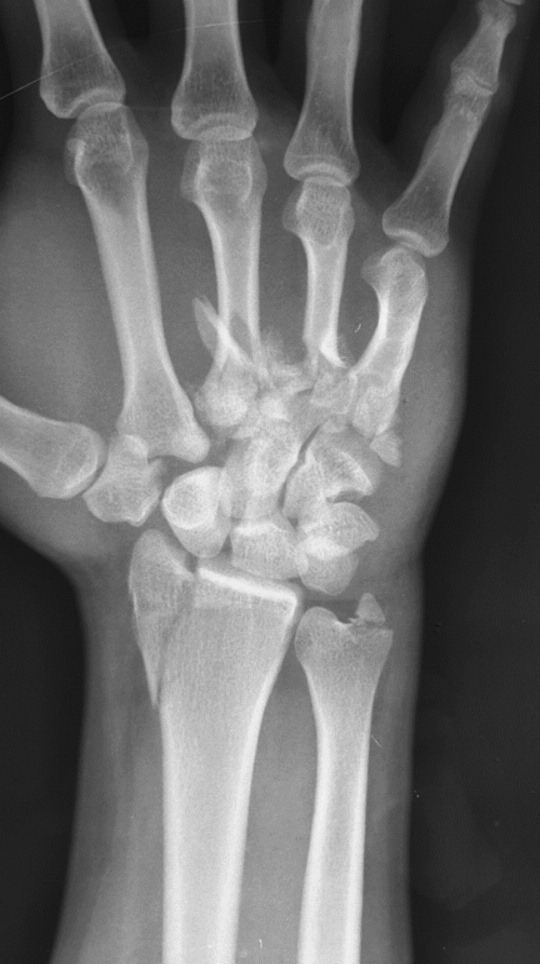
Radiograph shows the injuries of patient’s right hand.

A 23-year-old gentleman presented to us with swollen right hand after a motor vehicle accident. He was thrown from his motorcycle and landed on his right side. Radiographs revealed that there were fractures of the trapezoid, radial styloid, ulnar styloid, 3rd, 4th, and 5th metacarpal bones of the hand (Fig. 1).

He was hospitalised for a computerised tomography (CT) scan to further visualise the pattern of the fractures. However, before the CT scan, the injuries were complicated with compartment syndrome. Emergent fasciotomy was then performed. External fixator was also applied to temporarily immobilise the fractures and to assist in the post-operative wound care.

Due to the slightly prolonged wound care period post fasciotomy, definitive fixation was delayed. There was no CT scan performed after the first surgery.

**Fig. 2:**
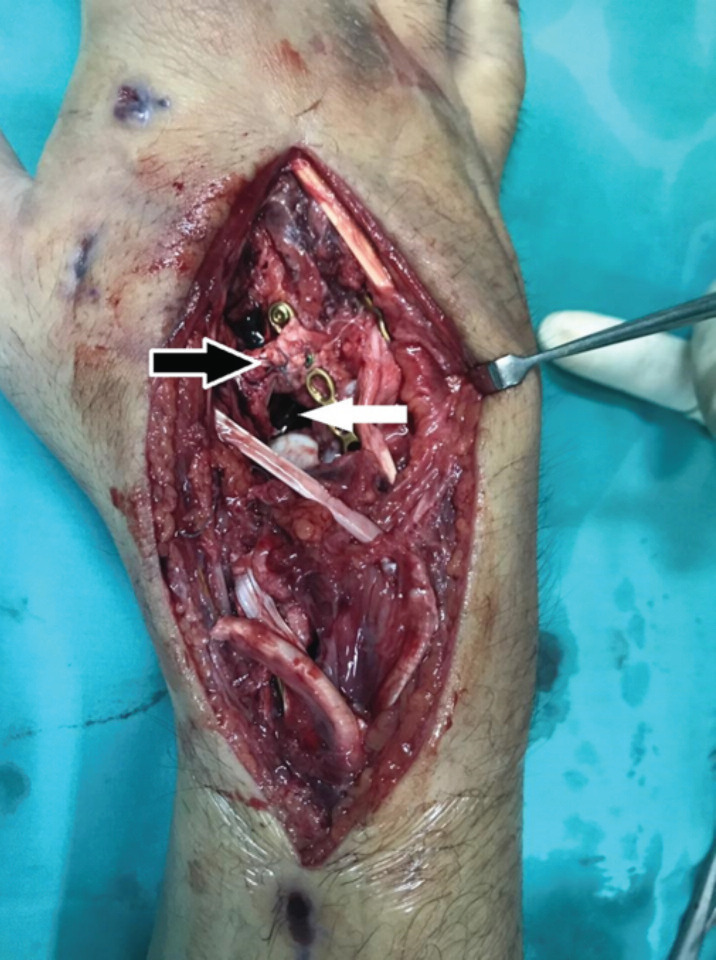
Black arrow shows that the ECRL is folded in anchovy-like fashion. White arrow shows the void which is to be filled with the folded ECRL.

During the definitive fixation, it was found that the trapezoid was comminuted and was completely extruded. There was no soft tissue (blood supply) attached to the bone. Excision of the trapezoid was performed. Distally based extensor carpi radialis longus (ECRL) was rolled up in an anchovy-like fashion to fill the void left by the trapezoid to achieve suspension interposition arthroplasty (Fig. 2).

**Fig. 3:**
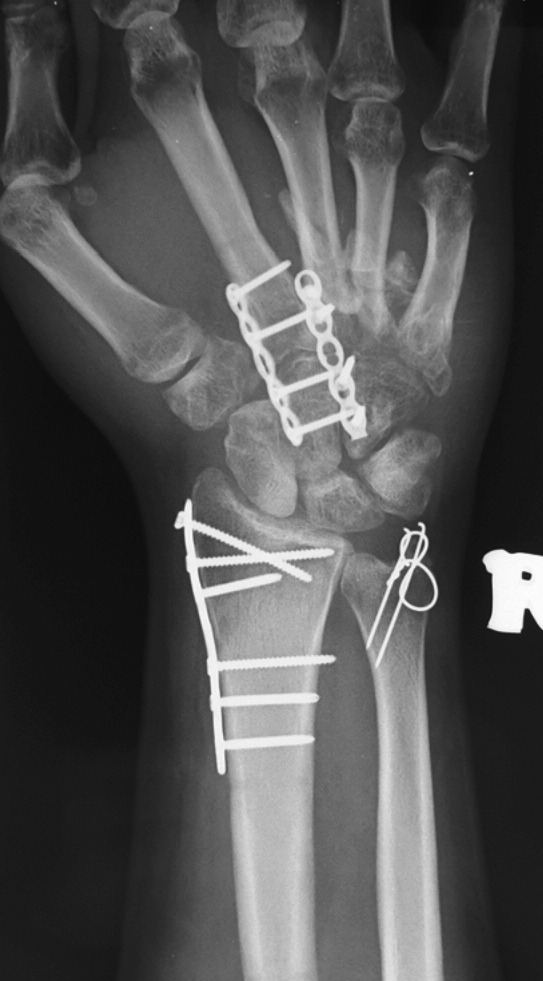
Radiograph shows the configuration of bones of patient's right hand after fixation.

There was a large quantity of callus at the fracture sites of the metacarpal bones. Due to the severe comminution of the base of 4th metacarpal bone, the base was not able to be reconstructed and was then excised. The 3rd metacarpal bone with a relatively intact base was shifted to the position of the 4th metacarpal bone. The 3rd metacarpal bone was plated to the hamate as shown in Fig. 3 [LCP Compact Hand, DePuy Synthes].

The distal fragment of the 4th metacarpal bone was not detached from the callus tissue. It was trimmed at the dorsal aspect to prevent prominence and was left in-situ to unite with the largely non-displaced 5th metacarpal bone.

The base of 2nd metacarpal bone was brought to articulate with the capitate and was plated as shown in Fig. 3 [LCP Compact Hand, DePuy Synthes].

The radial styloid was plated with no significant difficulty [LCP Distal Radius System 2.4, DePuy Synthes]. The ulnar styloid was fixed with tension band wire.

## Discussion

Trapezoid fractures consist of less than 1% of all carpal bone fractures^1^. Trapezoid and metacarpal bone form a very stable joint where dislocation of the joint is a rare event^3^. Carpometacarpal joint dislocation (in digits other than thumb) is uncommon because of the stable configuration of the joint^1^. This unfortunate patient had sustained all the injuries mentioned above. The motor vehicle accident was likely a high-energy trauma as evidenced by the fact that he was thrown from his motorcycle. The high-energy trauma caused severe injuries to both the bones and the soft tissues. The severe injuries ultimately developed into a compartment syndrome.

Severely comminuted trapezoid fracture which is beyond reconstruction is best managed by carpometacarpal arthrodesis because the motion between the trapezoid and the metacarpal bone is minimal^1^. However, CT scan was not performed for this patient due to the concern of expected artefact caused by the external fixator. The condition of the trapezoid was not fully visualised prior to the surgery; hence, bone grafting for arthrodesis was not included in the operative plan for this patient. With the experience gained from the current patient, removal of external fixator should be considered in future patients so that CT scan can play a role in the operative planning.

Total trapezoidectomy can cause proximal migration of the 2nd metacarpal bone^1^. However, in this patient’s specific situation, the utilisation of ECRL tendon was not aimed to prevent the proximal migration of the 2nd metacarpal bone as the metacarpal bone had already been plated in articulation with the capitate. It was utilised to prevent the collapse of the gap in between the trapezium and the capitate. This would further maintain the planes of the fingers and the thumb.

The base of 4th metacarpal bone is the keystone of the bony palmar arch. Power grip depends on the intact bony palmar arch^4^. Due to the severe comminution of the base of 4th metacarpal bone, the base was not able to act as the keystone of the arch. The 3rd metacarpal bone with a relatively intact base was shifted to the position of the 4th metacarpal bone. The 3rd metacarpal bone was then plated to the hamate to act as the new keystone. The 2nd metacarpal bone was transferred to articulate with the capitate proximally and with the 3rd metacarpal bone at the ulnar end to complete the arch [LCP Compact Hand, DePuy Synthes].

The fractures achieved union without complication. The Disability of Arm, Shoulder and Hand (DASH) questionnaire is an acceptable tool in evaluating the functions of wrist and hand with good reliability and reproducibility^5^. With proper post-operative rehabilitation, patient’s DASH score at the 6th month post-surgery was 35. He had mild difficulty in hand functions that require dexterity. Moderate difficulty was experienced by him in the hand functions that require power grip or heavy lifting. Removal of the implants was planned for this gentleman. Continuous rehabilitation is also needed for him in the future to further improve his hand functions after the removal of the implants.

The surgical management of this patient was mainly to aim for pain-free hand functions despite the severity of the injuries. The role of CT scan in surgery planning should not be overlooked. Principle-based intra-operative creativity and flexibility are an art to be cultivated in a surgeon.
